# Intracranial dural arteriovenous fistulas: A Review

**DOI:** 10.4103/0971-3026.45344

**Published:** 2009-02

**Authors:** AK Gupta, AL Periakaruppan

**Affiliations:** Department of Imaging Sciences and Interventional Radiology, Sree Chitra Tirunal Institute for Medical Sciences and Technology, Trivandrum - 695 011, India

**Keywords:** Coil embolisation, dural arteriovenous fistula

## Abstract

Dural arteriovenous fistulas are fistulas connecting the branches of dural arteries to dural veins or a venous sinus. Digital subtraction angiography remains the gold standard for diagnosing these fistulas. Endovascular treatment is one of the first line options available for their management. This review article reviews the etiopathogenesis, natural history, common classification systems and various available treatment options.

## Introduction

Yasargill noted that Rizzoli, in 1881, was the first to describe an arteriovenous malformation (AVM) that involved the dura mater and Sachs reported the first, angiographic description in 1931. Subsequently cranial dural fistulas have been most frequently described at the transverse sinus and cavernous sinus, although they occur at every cranial dural sinus. Dural arteriovenous fistulas (DAVFs) can occur anywhere within the intracranial dura mater. DAVFs are rare vascular abnormalities. They consist of numerous tiny connections between branches of dural arteries and veins or a venous sinus.[1] The true incidence of DAVFs is unknown.[2] However, the reported incidence of intracranial DAVFs is approximately 10-15% of all intracranial vascular abnormalities. A good percentage of DAVFs remain clinically silent or involute spontaneously and therefore the true incidence may be much more.[3] Present evidence suggests that DAVFs are acquired lesions and present later in life than AVMs.[4]

## Etiopathology

Before the mid-1970s DAVFs were thought to be congenital in origin and to be associated with other vascular lesions. In the late 1970s, Castaigne and Djindjian proposed an acquired etiology. They suggested that DAVFs may develop due to the opening up of existing micro shunts within the dura and by angioneogenesis, leading to the development of new shunts. Various etiologies are postulated: e.g., anomalies of venous system, venous thrombosis, head trauma, transcranial surgery, association with pregnancy, delivery and menopause, increased systemic thrombotic activity, cortical vein thrombosis (CVT), role of hormonal changes (use of oral contraceptive and pregnancy) causing increased angiogenesis, tumors (meningiomas obstruct dural venous outflow), general surgery, otitis, and sinusitis.[5]

Understanding of the pathophysiology of DAVFs came from the school of La Salpetriere, which emphasized the role of the cortical venous drainage of these dural shunts.[5] Henceforth, the role of the subarachnoid and subpial venous drainage could be correlated with the type of neurological manifestation and the natural history of dural shunts. Conditions associated with DAVFs include stenosis or occlusion of the draining dural sinuses, sinus thrombosis, prothrombin gene mutation, resistance to activated protein (APCR), mutation in factor V gene, and presence of antiphospholipid antibodies.

DAVFs in adulthood are an acquired disease and there is no relationship to hereditary or developmental disorders. On the other hand, we have seen brain AVMs as well as cavernomas occur in patients with DAVFs. Vascular diseases associated with dural arteriovenous shunts include cerebral AVM, maxillofacial AVM, hereditary hemorrhagic telangiectasia, bone AVM, cavernomas, and intradural or extradural arterial aneurysms.[5] Multiple DAVFs at separate locations in the same patient have also been reported.[5,6]

The common predisposing factor for DAVFs appears to be venous sinus thrombosis.[7] The venous hypertension developing after venous thrombosis opens up the microvascular connections within the dura.[8] Without intervention, these channels become hypertrophied resulting in direct shunting between the arteries and veins [Table 1].[7] When the fistula grows and becomes more diffuse, it recruits pial supply from parenchymal vessels. This may lead to the angiomatous network of multiple feeding arteries and numerous AV shunts within a partially recanalized sinus that is frequently seen at angiography. The involved dural sinus receives arterialized blood flow that can lead to mechanical obstruction of the sinus and result in retrograde drainage of blood away from the sinus and into the cortical veins. Histopathological analysis of excised DAVFs has revealed thrombosis and angiographic progression of sinus thrombosis to DAVF. In addition, dilatation of the cortical veins may occur, predisposing the patient to intracranial hemorrhage.

**Table 1:** Classification of dural arteriovenous fistulas

**Borden classification**	
1	Venous drainage directly into dural venous sinus or meningeal vein
2	Venous drainage into dural venous sinus with CVR
3	Venous drainage directly into subarachnoid veins (CVR only)
**Cognard *et al.***	
I	Venous drainage into dural venous sinus with antegrade flow
II a	Venous drainage into dural venous sinus with retrograde flow
II b	Venous drainage into dural venous sinus with antegrade flow and CVR
II a + b	Venous drainage into dural venous sinus with retrograde flow and CVR
III	Venous drainage directly into subarachnoid veins (CVR only)
IV	Type III with venous ectasias of the draining subarachnoid veins
V	Direct drainage into spinal perimedullary veins
	(CVR indicates cortical venous reflux)

Another mechanism for DAVF evolution is the release of angiogenic growth factors such as vascular endothelial growth factor (VEGF) and basic fibroblast growth factor (bFGF), which promotes neovascularization and development of a DAVF [Tables 2 and 3]. These factors by themselves should therefore not be considered as the direct cause of DAVFs, but rather they make up an environment that may be conducive to the development of DAVF, depending on the individual host response to their presence. DAVF in adults are triggered by factors that stimulate angiogenesis. If this angiogenic process is simultaneously associated with loss of some of the venular surface properties, then venous thrombosis is likely to occur. It has been noted that angiogenic growth factors such as basic fibroblast growth factor (bFGF) and vascular endothelial growth factor (VEGF) are expressed in the DAVFs. In DAVFs with a patent sinus, cortical venous pathways usually do not develop and hemorrhagic complications with these DAVFs are infrequent.[9]

## Osteodural-venous complex

At birth, the total cerebral venous drainage is directed towards the posterior sinuses, i.e. a true sinusal system.[5] Few months after birth that the so-called cavernous capture of sylvian vein will occur and offer the infant brain an alternate drainage via the cavernous plexus toward the orbit or the pterygoid plexus and into the external jugular venous system. The dura mater along the convexity of the cranial vault has a different origin from the dural along the base of the skull (membranous vs. cartilaginous bone). Septations may occur within the dural sinuses and result in separate venous channels, one of these channels may be used for the brain to drain while the other is sometimes exclusively used for the drainage of the DAVF and may be the specific target for treatment.[10] A peculiar group of lesions are the intracranial arteriovenous shunts fed by the dural arteries (that also supply the bone), which are located with in the bony structures along the convexity or at the base of the skull: the so called osteodural AVFs.

The incidence of DAVF at various locations as reported in literature is as follows: transverse sinus 50%, cavernous sinus 16%, tentorium cerebelli 12%, and superior sagittal sinus, 8%.[6]

## Natural history

The natural history of DAVFs is poorly documented and mostly reviewed retrospectively. Davis *et al*, followed 102 patients with intracranial DAVFs for an average of 33 months, with complete follow up in 91%. In his series, 55 patients had DAVFs without leptomeningeal and cortical venous reflux and, of these, 32 received no treatment. Among the patients who received no treatment, 81% had symptom improvement or complete resolution as compared to 86% of the treated patients with the same angioarchitecture. In the same series, there were 46 patients who had DAVFs with leptomeningeal or cortical venous reflux and 14 of these declined further treatment. At follow-up, this group had an 11% nonhemorrhagic neurological deficit rate per year and a 20% intracerebral hemorrhage (ICH) rate per year.[5] In an updated review of 118 patients with DAVFs and leptomeningeal reflux, the Toronto team demonstrated an annual risk for nonhemorrhagic neurological deficit of 6.9% and a risk of 8.1% for hemorrhage, with an annual mortality rate of 10.4%.[5]

Many DAVFs remain stable and do not change on follow-up. Some involute spontaneously; this may be due to thrombosis following DSA. Some DAVFs undergo progressive recruitment of pachymeningeal feeders with the worsening of symptoms. The actual progress of lesions once symptoms develop is unknown. By location, an aggressive course is seen in 75% of anterior fossa lesions, 79% of tentorial lesions, 60% of foramen magnum lesions, and 29% of transverse sinus lesions.[5] Factors predisposing to an aggressive course include leptomeningeal (cortical) venous drainage, galenic drainage (deep veins), variceal or aneurysmal venous dilatations, and location at the tentorial incisura. This incidence parallels that of the leptomeningeal retrograde venous drainage at each of these locations. The reported annual morbidity and mortality rates of aggressively presenting DAVFs may vary widely, ranging from 1.8-20% per year.[11]

## Clinical presentation

Patients with DAVFs may be completely asymptomatic. Symptoms, when present, may range from mild symptoms to fatal hemorrhage. The symptoms depend on the location and venous and drainage pattern of the DAVF. The clinical symptoms of the majority of DAVFs involving the superior sagittal, lateral, sigmoid and straight sinuses are often related to the route of venous drainage.[8] A petrous region DAVF draining into the transverse or sigmoid sinus commonly produces pulsatile tinnitus, with or without an associated bruit. Such patients may be managed conservatively, depending upon the pattern of venous drainage. Bruit and headache are common symptoms in DAVFs and are usually unrelated to cortical venous drainage. Carotid cavernous DAVFs often present with the classic clinical findings of proptosis, chemosis, and bruit. Treatment of a carotid cavernous DAVF is usually undertaken to protect against ocular and visual complications.

Symptoms of DAVFs may be characterized further as either nonaggressive (e.g., tinnitus) or aggressive (e.g., intracerebral, subarachnoid, or subdural hemorrhage and neurologic deficits).[5,10] These aggressive features are usually due to venous hypertension, although neurological deficits may be secondary to arterial steal.[6] A DAVF demonstrating venous occlusive disease is at a higher risk for complications from the arterialized collateral venous system. Aggressive neurological symptoms are more common in male patients who have aggressive angiographic features like retrograde leptomeningeal venous drainage, variceal or aneurysmal venous structures, and galenic venous drainage.[12] Earlier treatment of DAVFs that have these features is important to prevent complications. The risk of conversion from a benign to an aggressive DAVF is small but repeat angiography is indicated if the clinical picture appears to progress.[13]

## Classification system

The initial classification of DAVFs proposed in 1978 by Djindjian and Merland was based on venous drainage;[14] it was recently modified by Cognard *et al*,[15] Table 1 shows two commonly used classification systems. The Borden classification has three subtypes and is more user-friendly.[4] The Cognard system is based on venous drainage. It provides more detailed and elaborate information on the presence of normal or abnormal venous drainage and presence or absence of cortical venous recruitment and also takes into account the spinal perimedullary venous drainage.[10] Thus, it enables accurate comparison of clinical and radiological parameters.

## Imaging

Imaging by CT, CT angiography (CTA), MRI, and angiography play an important role in the investigation of patients with DAVFs. Due to varying nonspecific clinical and imaging features the diagnosis of a DAVF can be delayed or missed. Occasionally a plain film may be useful as it can show grooving within the skull vault due to chronic venous hypertension and compression by middle meningeal vessels.

Before any intervention, a nonenhanced CT must be done to rule out intracranial hemorrhage. Chronic venous congestion produces an area of low density on CT due to edema. Multidetector CTA, because of its rapid acquisition, can now provide high-resolution detail of vascular anatomy and has a temporal advantage over static CT. DAVFs can present as tinnitus and CT scan may help in detecting ear abnormalities such as aberrant vascular anatomy or glomus tumors. Linear bony defects formed by enlarged emissary veins can indicate the presence of a fistula.[16] MRI in DAVFs shows dilated cortical veins without a parenchymal nidus. It can also provide clues about DAVF in the presence of veno-occlusive disease. Other MRI imaging appearances include thickened dural leaflet; hypertrophied pachymeningeal arteries; dilated, tortuous, and variceal venous channels; and a thrombosed or stenosed dural venous sinus. The arterial supply to the DAVF is not demonstrated on MR images.

PET-based regional cerebral blood flow (rCBF) studies indicate that decreased rCBF may be important in the pathogenesis of the neurological symptoms. In patients with normal cortical venous drainage, values for rCBF, regional cerebral metabolic rate of oxygen (rCMRO), and regional oxygen extraction fraction (rOEF) were normal. Reduced rCBF and mildly or markedly increased rOEF is seen in patients with clinical symptoms and cortical venous drainage.[11]

## Angiography

Angiography remains the gold standard for diagnosis and planning of therapy. In addition to diagnosis, anatomical and hemodynamic assessment of the fistula needs to be performed. The aim is not only to identify the arterial feeders and the site of the fistula but also to identify the pattern and direction of venous drainage of the fistula and normal cerebral parenchyma. Selective injection of all potential arteries that may contribute to the region of dural pathology needs to be done. The acquisition of images should begin in the early arterial phase and continue into the late venous phase of the study. A dual arterial injection (competitive angiogram) may be required to study the venous drainage of fistula and normal brain. In this technique first selective injection is done in ICA followed by ECA. Then by using resubtraction and changing of the mask runs, drainage of the fistula and normal brain parenchyma can be superimposed. The angiogram should be analyzed for feeders, type of venous drainage, retrograde flow, occlusion of sinuses, and circulation time. The angiogram should be analyzed for feeders, type of venous drainage, retrograde flow, occlusion of sinuses, and circulation time. The common angiographic findings seen are feeders from pial and dural branches, AV shunting, venous ectasia, stenosis, calcifications, impaired drainage of cerebral parenchyma with delayed venous drainage, and cortical venous collaterals.

## Cortical rerouting

Aggressive symptoms are shown by those DAVFs, which drain retrogradely by leptomeningeal cortical venous channels. Awad *et al*, pointed out the aggressive factors as leptomeningeal cortical venous drainage, galenic deep venous drainage, variceal or aneurismal dilatations and lesions located at the tentorial incisura.[12] Houser *et al*,[8] point out that the sinus need not even be thrombosed to cause retrograde venous drainage into cortical veins. Partial filling of sinuses by the high-pressure arterial blood can impede antegrade venous drainage. A high frequency of hemorrhage is reported in DAVFs involving the anterior cranial fossa and tentorium. These DAVFs almost always drain into intradural veins.[17] Apart from hemorrhage, retrograde venous flow accompanied by increased venous pressure can lead to chronic passive congestion and venous infarcts, which are a major cause of focal neurologic deficits in DAVFs. Hydrocephalus is another complication of DAVF. It is due to decreased absorption of cerebrospinal fluid due to increased venous pressure or mechanical compression of the aqueduct by mesencephalic veins.

## Treatment

**Figure 1: (A-F):**
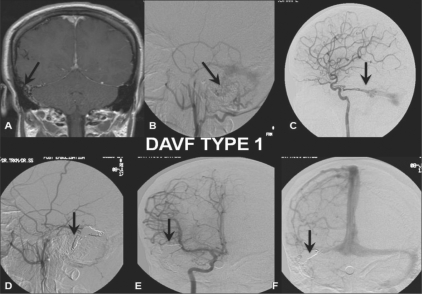
DAVF Type 1: Postcontrast coronal T1W MRI image (A) shows multiple tortuous flow voids (arrow) adjacent to the right sigmoid sinus. Selective right external carotid artery (ECA) (B) and internal carotid artery (ICA) (lateral view) (C) angiograms shows a DAVF type 1 with feeders (arrow) from the posterior meningeal branch of the middle meningeal artery and dural branches (arrow) from the cavernous ICA draining antegradely through the sigmoid sinus. Posttreatment selective right ECA (lateral view) (D) and ICA (anteroposterior view; arterial (E) and venous (F) phases) angiograms show coils (arrows) packing the right distal transverse and sigmoid sinus with total obliteration of the fistula

**Figure 2: (A-F):**
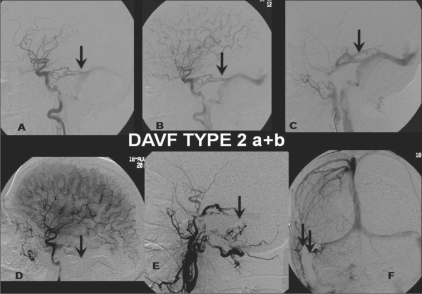
DAVF Type 2 a + b: Selective right ICA (A, B) and ECA (lateral view) (C) angiograms show a DAVF type 2 a + b, with feeders from the dural branches (arrow) of the ICA and the posterior meningeal branch of the middle meningeal artery (arrow) draining retrogradely through the transverse sinus. Post-treatment selective right ECA (lateral view) (D) and ICA (lateral view, arterial phase (E); anteroposterior view, venous phase (F)) angiograms show coils (arrows) packing the right distal transverse and sigmoid sinus with total obliteration of the fistula

**Figure 3: (A-F):**
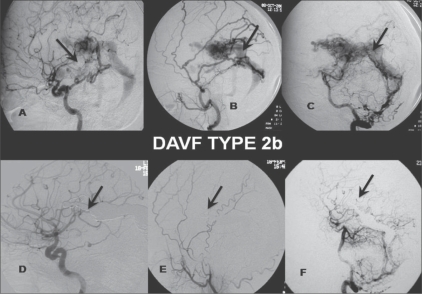
DAVF Type 2b: Selective right ICA (A), ECA (B) and vertebral artery (VA) (lateral view) (C) angiograms show DAVF type 2 b, with feeders from dural branches (arrow) of the ICA, posterior meningeal branch of the middle meningeal artery (arrow) and dural branches (arrow) from the V3 segment of the vertebral artery, draining antegradely through the straight sinus with venous reflux. Post-treatment selective right ICA (D), ECA (E), and VA (lateral view) (F) angiograms show coils (arrows) packing the vein of Galen and the straight sinus, with near-total obliteration of the fistula and cortical venous reflux

General treatment approaches for the treatment of DAVFs include the following:

1. Conservative treatment
2. Neuroradiological endovascular intervention, which comprises of:
  - Particulate embolization
  - Glue or onyx injection through arterial or venous route
  - Coiling with or without glue or onyx injection [Figures 1–3]
  - Venous stenting
3. Radiation therapy
4. Surgery
5. Combination of above, i.e., RT + intervention or intervention + surgery

### 1. Conservative treatment

Conservative treatment involves mainly symptomatic treatment and supportive measures. Spontaneous regression of dural DAVFs has been reported.[18]

### 2. Endovascular neuroradiological intervention

#### Transarterial route

- *Particulate embolization*: Embolization of external carotid branches with particles is easily performed and can reduce shunt flow. Transarterial particulate embolization typically is not curative. Total obliteration is difficult to achieve with this method because some feeding arteries cannot be catheterized and because of the recruitment of blood supply from collateral arteries.[19] Therefore, it is generally used to relieve symptoms or in combination with other procedures such as irradiation, surgery, or transvenous embolization (TVE).
- *N-butyl-2-cyanoacrylate or onyx embolization*: Deposition of glue into the collecting vein or fistula through the transarterial route may also result in cure, but it is less controllable than coil embolization and thus may present a higher risk. Transarterial embolization (TAE) with N-butyl-2-cyanoacrylate is regularly used to treat complex DAVFs if percutaneous transvenous catheterization is not possible.[20] Onyx is a new nonadhesive liquid embolic agent. It is supplied in ready-to-use vials and is a mixture of EVOH, DMSO, and tantalum. Onyx, after coming in contact with blood, gets precipitated and occludes the vessels but is nonadherent to the vessel wall. This non-adhesive property of onyx eliminates the risk of gluing of the microcatheter to the vessels. This also allows a slow and prolonged single injection of the embolic agent with concomitant check angiograms. Good positioning of the microcatheter and slow injection can completely fill the fistula and enable a complete cure.
  Currently onyx is available in two concentrations (FDA-approved) in the United States for presurgical cerebral AVM embolization.[21] Depending on the need, onyx or glue in mild to moderate concentration (20, 25, or 33%) may be injected.
- *Coiling via transvenous route*: Coils are now regularly used for curative purposes. Many studies have reported their usefulness. Complete occlusion may occur in 80-100% of cases.[22] An angiographically nonvisualized inferior petrosal sinus (IPS) can be successfully catheterized in 30-50% of patients.[9] When this is thrombosed, the transvenous access may be tried out via the superior petrosal sinus (SPS) facial vein, angular vein, pterygoid sinus, and superior ophthalmic vein (SOV)[23] Inadequate embolization leads to a worsening of symptoms.
  The use of detachable coils before onyx or glue injection slows and decreases flow in the fistula and provides secure anchoring to the onyx or glue cast.
- Stent placement: Some authors have tried to close the shunts in the dural arteriovenous fistulas with the help of stents.[24] Kieran *et al*, have postulated that it is possible to recanalize a chronically occluded dural venous sinus and complete closure of multiple DAVF feeders can be achieved by the deployment of stents.[25] Long-term results from the use of stents in the treatment of DAVFs are not yet known. Antegrade sinus flow can be established with the help of the radial force in the stent, which can close the shunts in the sinus wall.[24]

### 3. Radiation therapy

Good results have been reported from the use of stereotactic radiotherapy for the treatment of DAVFs, with complete occlusion being reported in 44-87% of cases.[26,27] This method, although reported to be effective in several case reports or small series, carries an unacceptable delay of 1–3 years in the cure of DAVFs with cortical venous reflux (CVR) and therefore is not recommended as a primary therapeutic measure for these lesions.[28] The advantages of radiotherapy include decreased invasiveness of the procedure and fewer short-term complications; the major disadvantage is the latency of 6–12 months after irradiation before there are tangible benefits. The combined use of stereotactic radiotherapy and TAE with particles is in practice and it enhances the effectiveness of both the techniques. Combined treatment gives good results and reduces the risk of worsening symptoms during the follow-up period.[29,30]

### 4. Surgery

Endovascular management has become a first-line treatment for dural DAVFs. However, some DAVFs of the anterior cranial fossa can often be treated more easily and safely with surgical disconnection of the venous drainage.[29] Combination with endovascular procedures is required in difficult cases where sinus isolation and resection are needed.

## Conclusions

DAVFs with retrograde leptomeningeal venous drainage carry a high risk for neurological sequelae or death, both at presentation and in the natural course of disease progression. With presently available treatment modalities most of these lesions are either curable or, at the very least, patients may get significant clinical improvement. The natural history of the condition suggests that these lesions are aggressive and need prompt diagnosis and treatment.
